# Prevalence of Occult Hepatitis C Virus in Egyptian Patients with Chronic Lymphoproliferative Disorders

**DOI:** 10.1155/2012/429784

**Published:** 2012-12-12

**Authors:** Samar Samir Youssef, Aml S. Nasr, Taher El Zanaty, Rasha Sayed El Rawi, Mervat M. Mattar

**Affiliations:** ^1^Microbial Biotechnology Department, National Research Centre, Cairo 12311, Egypt; ^2^Department of Clinical Pathology, Faculty of Medicine, Cairo University, Giza 12613, Egypt; ^3^Department of Internal Medicine, Faculty of Medicine, Cairo University, Giza 12613, Egypt

## Abstract

*Background.* Occult hepatitis C virus infection (OCI) was identified as a new form of Hepatitis C virus (HCV), characterized by undetectable HCV antibodies and HCV RNA in serum, while HCV RNA is detectable in liver and peripheral blood cells only. *Aim.* The aim of this study was to investigate the occurrence of OCI in Egyptian patients with lymphoproliferative disorders (LPDs) and to compare its prevalence with that of HCV in those patients. *Subjects and Methods.* The current study included 100 subjects, 50 of them were newly diagnosed cases having different lymphoproliferative disorders (patients group), and 50 were apparently healthy volunteers (controls group). HCV antibodies were detected by ELISA, HCV RNA was detected in serum and peripheral blood mononuclear cells (PBMCs) by reverse transcription polymerase chain reaction(RT-PCR), and HCV genotype was detected by INNO-LiPA. *Results.* OCI was detected in 20% of patients group, compared to only 4% OCI in controls group. HCV was detected in 26% of patients group with a slightly higher prevalence. There was a male predominance in both HCV and OCI. All HCV positive patients were genotype 4. *Conclusion.* Our data revealed occurrence of occult HCV infection in Egyptian LPD patients at a prevalence of 20% compared to 26% of HCV.

## 1. Introduction

Chronic hepatitis C virus (HCV) infection remains a global health threat with 175 million carriers worldwide. Approximately 3% of the worldwide population is infected with the hepatitis C virus (HCV) [1]. The prevalence of HCV infection varies throughout the world, with the highest prevalence reported in Egypt [2]. HCV is an RNA virus that belongs to the family of flaviviruses [3]. The natural targets of HCV are hepatocytes and, possibly, B lymphocytes [4, 5].

A new form of chronic HCV infection named occult hepatitis C virus (OCI) was described by Castillo et al., 2004 [6]. This infection is characterized by absence of anti-HCV antibodies (Abs) and HCV RNA in serum with elevated liver function tests in the presence of HCV-RNA in the liver [6]. Furthermore, about 70% of patients with occult HCV infection also have HCV RNA in their peripheral blood mononuclear cells (PBMCs); the genomic and the antigenomic HCV RNA have also been detected in these cells [7]. Although detection of genomic HCV-RNA strand in liver biopsy specimen is the gold standard and the most accurate method for the diagnosis of occult HCV infection, testing for HCV-RNA in PBMCs is an alternative and easy to do when a liver biopsy is not available [8, 9].

Lymphoproliferative disorders is a term that includes a wide spectrum of pathologies ranging from a minor expansion of a B-cell population (with no clinical significance) to an aggressive high-grade lymphoma. Such proliferations of B cells apparently can be triggered as a consequence of a chronic antigenic stimulation resulting from an HCV infection [10]. Non-Hodgkin lymphoma (NHL) is the hematologic malignancy with the highest prevalence worldwide. Incidence rates have grown fast up to the beginning of the new millennium, with an annual percentage increase of nearly 3%, which is faster than that for most cancers [11].

A causative association between hepatotropic viruses, especially hepatitis C virus, and malignant B-cell lymphoproliferative disorders has been demonstrated utilizing epidemiologic data, biologic and molecular investigations, as well as clinical observations. These data indicate that hepatitis C virus may be responsible for the development of some malignant lymphoproliferative disorders [11–17].

A former study on the malignant complications of chronic HCV infection in Egypt and association of HCV with increased risk of B-cell NHL as a whole was reported [18]. Recently, another study in Egypt proved that HCV is a risk factor for diffuse large B cell, marginal zone, and follicular lymphomas in Egypt [19]. Nonetheless, assessment of the existence of occult hepatitis C infection in LPD patients has not been addressed in Egypt and worldwide till now.

The objective of this study was to investigate the occurrence of occult Hepatitis C infection in lymphoproliferative disorders patients and to compare its prevalence with that of HCV in those patients.

## 2. Subjects and Methods

### 2.1. Subjects

All subjects included in the study were obtained from Clinical Hematology Unit of Internal Medicine Hospital at Kasr El-Eini School of medicine, Cairo University in the period between June 2010 and June 2011. Institutional ethical board approval was taken prior to the study, as well as informed consent was taken from all the participants. A total of 100 subjects were included in this study. Fifty of them were consecutive newly diagnosed LPD patients. Selection criteria for patients were to being confirm LPD patients, negative for infection with hepatitis B virus (HBV), HIV, Epstein-Barr virus (EBV), and cytomegalovirus (CMV). Out of the recruited 50 patients, there were 20 patients with non-Hodgkin lymphoma, 4 patients with Hodgkin disease, and 26 patients with chronic lymphocytic leukemia. The other 50 subjects were apparently healthy volunteers who were selected to be negative for HCV Ab and serum HCV RNA and to be age and sex matched with LPD patients recruited.

All the subjects included in the study were subjected for full history taking and clinical examination, complete blood picture (CBC) with differential white cell count, complete liver and kidney functions, and HCV antibodies using the commercially available ELISA kits (Dia Sorin, Torino, Italy).

The patients group was subjected as well to beta 2 microglobulin, abdominal ultrasound for detection of organomegaly and lymphadenopathy, CT abdomen and pelvis for proper diagnosis and staging, immunophenotyping to role out chronic lymphocytic leukemia, lymph node biopsy for diagnosis of non-Hodgkin lymphoma and bone marrow biopsy, and immunohistochemistry for staging.

### 2.2. HCV Antibodies Detection and HCV Genotyping

Serum HCV antibodies were detected by ELISA (Dia Sorin, Torino, Italy), while HCV genotyping was detected by INNO-LiPA HCV II (Bayer Health Care, Eragny, France).

### 2.3. RNA Extraction from Serum and PBMC

RNA was extracted from serum and PBMC using Biozol (Bioflux, China, Catalogue no. BSJ000210001 M80) total RNA extraction reagent according to the manufacturer's protocol.

### 2.4. Detection of the HCV RNA Plus Strand

HCV RNA plus strand was determined by reverse transcription-polymerase chain reaction (RT-PCR). RNA was reverse-transcribed and amplified by One Step RT-PCR QIAGEN Kit (Catalogue no. 210212, sensitivity 22 viral copies) with appropriate primers (5′-CGC GCG ACT AGG AAG ACT TC-3′) and (5′-ATA GAG AAA GAG CAAC CA GG-3′) as forward and reverse primers, respectively. The lack of contamination in PCR reactions and was assessed by the inclusion of a negative control containing water rather than RNA in each assay which did not show any PCR amplification in all experiments. Moreover, each sample was done in duplicate to ensure absence of false positive results. Thermal cycling conditions were denaturation: for 1 min at 94°C, annealing: for 1 min at 55°C, extension: for 1 min at 72°C for 30 cycles, and final extension for 10 min at 72°C. The PCR product (174 bp) was submitted to electrophoresis by using a 1.5 agarose gel and was visualized by ethidium bromide staining under ultraviolet light.

### 2.5. Detection of the HCV Minus Strand in LPD Patients with OCI

HCV RNA minus strand was determined by a previously established and described in house RT-PCR assay [20]. Reverse transcription was performed in 25 *μ*L reaction mixture containing 20 U of AMV reverse transcriptase (Clontech, USA) with 400 ng (3 *μ*L) of total PBMCs RNA, 40 U of RNasin (Clontech, USA), 0.2 mmol/L from each dNTP (Promega, Madison, WI, USA), and 50 pmol of the forward primer 2CH (for minus strand). The reverse transcription reaction was performed at 42°C for one hour. Amplification of the highly conserved 5′-UTR sequences was done using two PCR rounds with two pairs of nested primers. First round amplification was done in 50 *μ*L reaction mixture, containing 50 pmol from each of 2CH (5′-AAC TAC TGT CTT CAC GCA GAA-3′) forward primer and P2 (5′-TGC TCA TGG TGC ACG GTC TA-3′) reverse primer, 0.2 mmol/L from each dNTP, 10 *μ*L from RT reaction mixture as template, and 2 U of Taq DNA polymerase (Promega, USA) in a 1x buffer supplied with the enzyme. A positive control RNA of an HCV patient previously tested and confirmed to harbor that only the negative strand was included. Moreover, two types of negative controls were included, a negative RT control having no RNA at the reverse transcription step and a PCR negative control having water instead of cDNA. The thermal cycling profile was 1 min at 94°C, 1 min at 55°C, and 1 min at 72°C for 30 cycles. The second round amplification was done similar to the first round, except for use of the nested reverse primer D2 (5′-ACT CGG CTA GCA GTC TCG CG-3′) and forward primer F2 (5′-GTGCAGCCTCCAGGACCC-3′) at 50 pmol each. PCR (179 bp) products were analyzed on 2% agarose gel electrolysis.

## 3. Statistics

Statistical calculations were performed using Microsoft Excel version 7 (Microsoft Corp., Redmond, WA, USA) and SPSS for Windows version 16 (SPSS Inc., Chicago, IL, USA) software.

Results were reported as mean ± standard deviation (±SD) or frequency (%) when appropriate. Comparison of quantitative variables between the study groups was done using one-way analysis of variance test. Correlation between various variables was done using Pearson moment correlation equation for linear relation. Correlation was considered large if between 1.0 and 0.5, medium if between 0.5 and 0.3, weak if between 0.3 and 0.1, and no correlation if between 0.1 and 0.0. *P* value less than 0.05 was considered statistically significant and less than 0.01 was considered statistically highly significant.

## 4. Results

### 4.1. Demographic, Clinical, and Laboratory Results

The demographic and clinical data of the LPD patients group are summarized in Table 1, also there were no statistically significant differences between the 2 groups regarding sex (*P* = 0.0639) and age (*P* = 0.25). There were highly statistically significant differences between the 2 groups regarding hepatomegaly, splenomegaly, and lymphadenopathy (*P* < 0.0001). The laboratory data of the patients and control are shown in Table 2. The laboratory data of the patients and controls are shown in Table 2, and high statistical significance differences were seen between the 2 studied groups in haemoglobin, platelet count, AST, and ALT (*P* < 0.0001).

### 4.2. Results of HCV Ab and Plus and Minus HCV Strands Detection

**Figure 1:**
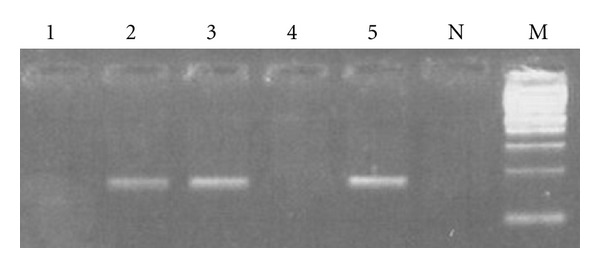
Results of PCR amplification of HCV plus RNA strand in PBMC. PCR products of amplification of HCV plus strand from PBMCs of LPD patients (lanes 1, 2, 3, and 4). Lane N represents the negative control of the PCR. Lane 5 is the positive control of the PCR. Lane M is 100 Bp Mwt marker.

In LPD patients, HCV Ab was detected in 13 out of 50 (26%) patients, and serum HCV RNA detection results were identical to HCV Ab positivity, so 13 out of 50 (26%) patients were positive. PBMC HCV plus strand was detected in 18 out of 50 (36%) LPD patients (Figure 1), and among these, ten (20%) patients were negative for HCV Ab and serum HCV RNA representing OCI patients. PBMC HCV minus strand was checked in the ten OCI positive LPD patients only and was undetectable in all of them. In controls group PBMC HCV plus strand was detected in only 2 out of 50 cases (4%) representing OCI prevalence in this group. Statistical analysis showed significant differences between patients and controls regarding the incidence of occult HCV (*P* = 0.0001), suggesting that occult HCV is associated with NHL and can be considered as a risk factor for NHL. RT-PCR results for the detection of HCV minus strand in PBMC of OCI positive patients were all negative.

### 4.3. HCV Genotyping Results

HCV genotyping results showed that all HCV and OCI positive patients were genotype 4.

### 4.4. Correlation of Clinical and Laboratory Data of HCV Positive LPD Patients, OCI Positive LPD Patients, and HCV Negative OCI Negative LPD Patients Groups

As shown in Table 3, according to Pearson correlation, there were no correlations between age, hepatosplenomegaly, sex, TLC, AST, and total bilirubin, while there was positive correlation between Hb, platelet count, ALT, and positive HCV antibodies LPD patients. In OCI positive LPD patients, there were no correlations between age, hepatosplenomegaly, sex, Hb, TLC, and ALT, while there was positive correlation between platelet count, AST, total bilirubin, and OCI positive patients. In HCV negative OCI negative LPD patients groups, there was no statistically significant correlation with age, hepatosplenomegaly, sex, TLC, AST, Hb, platelet count, ALT, and total bilirubin. In both HCV positive and OCI positive patients, number of male patients exceeded that of female patients. Among ten OCI positive patients, six were males, while nine of thirteen HCV patients were males.

### 4.5. Results of Association between Blood Transfusion and HCV Positivity

In order to identify the association between blood transfusion and HCV positivity in LPD patients, we identified the total number of patients subjected to blood transfusion. Out of 50 LPD patients, 16 patients received blood transfusion including 5 anti-HCV positive LPD patients, 5 OCI positive LPD patients, and 6 LPD patients negative or anti-HCV and OCI. Subsequently, statistical analysis was used to assess this association (Table 4). There was statistically significant difference between the total number of HCV (HCV + OCI) positive LPD and non-HCV positive LPD patients and the blood transfusion, proving positive role of blood transfusion in LPD patients having HCV over those without HCV.

## 5. Discussion

Multiple reports have described an association between chronic HCV infection and B-cell NHL [21]. One of those concluded that the prevalence of HCV infection in patients with B-cell NHL was 15% much greater than in the general population [22]. In Egyptian population, this association was also proved by Goldman et al., 2009 [23].

Occult HCV infection (OCI) is a new entity of HCV. Authors have long struggled to prove the existence of occult hepatitis C infection (OCI). Recently, it has been documented in haemodialysis patients, in chronic HCV patients after SVR, in general populations, and in chronic liver disease patients of unknown etiology [24–28]; nonetheless, it have never been studied in LPD patients. In Egypt, a recent study have shown high incidence of OCI in nonalcoholic liver disease (40.7%) [29]. To the best of our knowledge, this study is considered the first to investigate the detection of OCI in LPD patients.

Surprisingly, our results showed the detection of OCI in 20% of NHL patients compared to 4% OCI only in control healthy volunteers. Statistical analysis of the differential existence of OCI in LPD patients versus healthy controls showed a highly significant *P* value equal to 0.0312, confirming association of OCI with LPD and suggesting its incrimination in lymphomagenesis. It should be noted that detecting OCI by testing HCV RNA in PBMC is an alternative method when liver biopsy is not available, and that OCI is detected in PBMC of 70% only of patients with OCI. Therefore, it should be expected that the prevalence of OCI in LPD patients might exceed that reported in this study if detected in liver biopsies.

In this study, 4% of control healthy volunteers were OCI positive, which is the first record for the prevalence of OCI in healthy individuals in Egypt. This is consistent with that recorded by De Marco et al., 2009 [25], which showed evidence that occult HCV infection may occur in 3.3% of population unselected for hepatic disease.

In the present study, the patients selection criteria were to be newly diagnosed LPD patient and was blind regarding positivity for HCV antibodies (Abs)s in order to compare the prevalence of HCV with that of OCI blindly. Results showed HCV prevalence of 26%, which is slightly higher than OCI (20%). Consequently, the total burden of HCV in LPD patients calculated based on the existence of HCV only versus existence of both OCI and HCV was raised from 26% to 52%, respectively.

The HCV genotype detected in LPD patients positive for either HCV or OCI was genotype 4. This is consistent with previous studies reporting predominance of this genotype in Egypt [30].

Previous studies indicated that 22–66 years is the common age of OCI. Our results agreed with that. Moreover, in our study, OCI was detected in a young male patient 18 years old, reflecting its occurrence in such young age. This, together with the fact that male sex is predominant in OCI is accordant with previous studies by [7] and [31].

Saad et al. [29] reported that occult HCV infection seems to induce a mild liver disease, and Followup is recommended for the occult HCV patients to monitor progression to overt disease. Accordingly, it should be taken into consideration that NHL patients with elevated liver function tests with unknown etiology should be tested for OCI and should be carefully followed up.

Blood transfusion was examined as a risk factor for occurrence of HCV and OCI in LPD patients. As shown in the Results section, statistical analysis of its role in LPD HCV Ab positive patients alone was not significant and similarly for LPD OCI alone was not significant. Nonetheless a significant correlation was recorded when calculation was based on the total HCV plus OCI, raising the inevitable role of OCI in studying this risk factor. On the other hand, it should be noted that OCI positive NHL patients who did not receive blood transfusion are equal in number with those who received it, indicating that there are two accepted probabilities, either HCV and OCI can be acquired in LPD patients as a consequence of blood transfusion of infected blood, or LPD may arise as a disorder due to HCV infection.

In conclusion, the present study, although having relatively low number of patients, is the first to demonstrate the occurrence of OCI in LPD patients in Egypt and worldwide and to highlight the fact that the burden of HCV in LPD is doubled if OCI is considered. Further studies with larger sample are recommended in order to assess the impact of OCI on LPD progression.

## Figures and Tables

**Table 1:** Demographic and clinical data of patients and controls groups.

	Patients (*N*) %		Controls (*N*) %		*P* value
Sex					
Female	24 (48%)		14 (28%)		0.0637*
Male	26 (52%)		36 (72%)		
Hepatomegaly	25 (50%)		4 (3%)		<0.0001***
Splenomegaly	42 (84%)		4 (3%)		<0.0001***
Lymphadenopathy	27 (54%)		2 (1%)		<0.0001***
Age (years)	Range	Mean ± SD	Range	Mean ± SD	
	18–68	45.8 ± 12.7	20–63	38.2 ± 11.8	0.25*

*N*: number of subjects. *Not significant (NS) (*P* value > 0.05), **significant (S) (*P* value < 0.05), ***highly significant (HS) (*P* value < 0.0001).

**Table 2:** Laboratory data of patients and controls groups.

	Patients (*N* = 50)		Controls (*N* = 50)		*P* value
	Range	Mean ± SD	Range	Mean ± SD	
Hemoglobin (gm%)	6.5–16	10.6 ± 2.5	11.4–16	13.3 ± 1.4	<0.0001***
Platelet count ×10^3^ mm³	21–567	172.7 ± 104.8	168–394	282.5 ± 63.5	<0.0001***
Total leucocytic count/mm³	1.2–33	8.6 ± 5.6	6–15	10.0 ± 2.4	0.1074*
AST (IU/dL)	11–192	41.9 ± 38.0	10–38	15.7 ± 2.1	<0.0001***
ALT (IU/dL)	7–133	34.1 ± 29.9	12–48	18 ± 3.4	<0.0001***
Bilirubin (mg/dL)	0.10–2.80	0.75 ± 0.64	0.1–1.1	0.8 ± 0.2	0.5992*

*N*: number of subjects. *Not significant (NS) (*P* value > 0.05), **significant (S) (*P* value < 0.05), ***highly significant (HS) (*P* value < 0.0001).

**Table 3:** Correlation between clinical and laboratory data in HCV positive and OCI positive LPD patients versus HCV and OCI negative LPD patients.

Parameter	LPD patients					
	HCV positive		OCI positive		HCV and OCI negative	
	*r*	*P* value	*r*	*P* value	*r*	*P* value
Age	0.091	0.528	0.082	0.571	0.018	0.9
Hepatomegaly	0.060	0.267	0.063	0.257	0.161	0.244
Splenomegaly	0.092	0.526	0.089	0.538	0.095	0.514
Sex	0.022	0.399	0.096	0.172	0.0171	0.234
Hb (gm%)	0.323	0.037	0.086	0.552	0.092	0.525
Platelets ×10^3^ mm³	0.437	0.040	0.316	0.013	0.065	0.653
TLC/mm³	0.074	0.608	0.018	0.413	0.000	0.999
AST	0.022	0.398	0.446	0.053	0.048	0.742
ALT	0.221	0.044	0.029	0.110	0.250	0.08
Bilirubin (T)	0.059	0.682	0.518	0.021	0.236	0.098

**Table 4:** Comparison of different patients group regarding blood transfusion.

Blood transfusion					
HCV, not occult = +ve (abs & PCR)	Occult HCV	*P* value	Total HCV	Total non-HCV	*P* value
(31.25%) 5/16	(31.25%) 5/16	0.987	(62.5%) 10/16	(37.5%) 6/16	0.0214
